# Effect of biotin supplementation and swimming training on oxidative stress and testicular function in male rats

**DOI:** 10.1016/j.heliyon.2025.e42468

**Published:** 2025-02-04

**Authors:** Shadi Almasi, Mohammad Reza Jafarzadeh Shirazi, Mohammad Reza Rezvani, Mahdi Ramezani, Iraj Salehi, Sasan Javid-Moghadam, Alireza Komaki

**Affiliations:** a Department of Animal Science, School of Agriculture, Shiraz University, Shiraz, Iran; b Department of Anatomy, School of Medicine, Hamadan University of Medical Sciences, Hamadan, Iran; c Department of Neuroscience, School of Science and Advanced Technologies in Medicine, Hamadan University of Medical Sciences, Hamadan, Iran; d Department of Physical Education and Sport Sciences, Hamedan Branch, Islamic Azad University, Hamedan, Iran

**Keywords:** Biotin supplementation, Infertility, Swimming training, Testes

## Abstract

Oxidative stress (OS) affects testicular function and is a significant cause of sperm cell dysfunction in males. The development of male infertility is closely linked to a sedentary lifestyle and diet. This study aimed to characterize the protective effects of biotin supplementation (BS) and swimming training (ST) on OS markers in the reproductive system of male rats. Forty male rats (200–250 g) were randomly assigned to four groups (n = 10 per group) and treated for 28 days as follows: control, BS (this group received BS through oral gavage), ST, and BS + ST groups. Our results showed that glutathione (GSH) levels significantly increased in the BS, ST (p < 0.05), and BS + ST (p < 0.01) groups, whereas malondialdehyde (MDA) levels significantly decreased in these experimental groups (p < 0.05 for all). Additionally, compared to the controls, there was a significant increase (p < 0.05) in blood levels of follicle-stimulating hormone (FSH), luteinizing hormone (LH), testosterone (T), and *high-density lipoprotein* (HDL) in the BS and ST groups evidenced by a decrease in the levels of other biochemical parameters (cholesterol (CHO), triglyceride (TG), low-density lipoprotein (LDL), *alanine transaminase* (ALT), aspartate transaminase (AST), and alkaline phosphatase (ALP)). According to histological examination, the BS, ST, and BS + ST groups showed an improvement compared to the control group. In conclusion, BS had positive effects on biochemical parameters and antioxidant activity, and BS along with ST improved testicular function in male rats.

## Highlights

• Biotin has antioxidant properties.
• Oxidative stress has been suggested as an inducer for infertility.
• Biotin attenuate the effects of oxidative stress on infertility.
• Biotin is a potential neuroprotective agent against infertility.

## 1 Introduction

In animal research, a decrease in horse sperm motility was observed in relation to reactive oxygen species (ROS) [1]. Subsequent studies by other researchers have demonstrated that elevated ROS levels contribute to the reduction in motility and viability of bovine sperm cells [2]. As a result, oxidative stress (OS) has emerged as a key area of interest as a potential factor in male infertility [3]. Excessive ROS can induce OS, leading to lipid peroxidation (LPO) and causing damage to the viability of sperm, as well as other testicular cells' deoxyribonucleic acid (DNA), ribonucleic acid (RNA), and proteins [4]. Mitochondria play a crucial role in producing ROS within sperm cells, primarily through oxidative phosphorylation [5]. The performance of sperm mitochondria is closely tied to the motility of these cells. Therefore, any disturbance in the balance between oxidative and antioxidant mechanisms may impede sperm motility by affecting the function of sperm mitochondria [6]. Furthermore, ROS can directly damage sperm DNA. Since sperm cells lack the cytoplasmic enzyme systems required for the molecular processes involved in DNA repair, they are unable to efficiently repair damaged DNA [7]. Biotin, a water-soluble vitamin, serves as a coenzyme for four carboxylases: pyruvate carboxylase (PC), acetyl-CoA carboxylase (ACC), propionyl-CoA carboxylase (PCC), and methylcrotonyl-CoA carboxylase (MCC) [8]. These enzymes play a vital role in diverse metabolic processes including fatty acid synthesis, branched-chain amino acid catabolism, and gluconeogenesis, which are essential for cellular energy generation and storage [9]. In cases of biotin deficiency, MCC accumulates in the mitochondria, resulting in a decrease in succinyl-CoA (SC) and glycine levels. This deficiency impedes the synthesis of heme/cytochrome, leading to the generation of mitochondrial ROS and premature cellular senescence [10]. Biotin plays a crucial role in regulating gene expression through histone biotinylation. Biotinylation is also affected by insufficient biotin levels [11]. These factors can impact cellular proliferation, gene suppression, cellular apoptosis, and DNA repair mechanisms through the apoptosis process [12]. It also counteracts the impact of numerous naturally occurring free radicals in the body [13]. Biotin supplementation (BS) in the sperm preparation medium has the potential to enhance sperm motility and prolong the viability of frozen-thawed semen samples, potentially aiding in reproductive advancements [14]. This suggests that addressing biotin deficiency can aid in mitochondrial metabolism, facilitating ATP production [15,16]. However, elevated biotin levels may inhibit sperm production [17]. Numerous studies have explored the link between exercise training (ET) and fertility using animal models [13]. Swimming in small laboratory organisms has been extensively employed to study physiological alterations [18]. A recent study investigated the influence of swimming on the male reproductive system in rats treated with isoproterenol, a compound known to trigger oxidative damage in the testes. The detrimental effects of heightened OS were successfully alleviated through participation in moderate-intensity swimming training (ST) [13]. OS can lead to reduced swimming performance in current models [15]. Furthermore, severe OS induced by swimming can impede male fertility [19]. However, moderate swimming activity serves a protective function against OS and testicular stimulation, while also influencing the ratio of mature to young sperm in a rodent model [20]. ROS are critical for the immune system's defense mechanisms against pathogens, as they play a key role in microbial destruction. However, excessive oxidative stress can negatively impact leukocytes by damaging their cellular structures, including membranes and DNA, which can compromise their functionality and lead to inflammation. Leukocytes themselves are a source of ROS, but they are also highly susceptible to ROS-induced damage [21]. Overproduction of ROS by leukocytes and immature spermatozoa (SZ) can lead to LPO and DNA damage in healthy SZ, further exacerbating inflammatory responses [22,23]. To maintain the integrity and functionality of leukocytes, antioxidants are crucial. These molecules protect leukocytes from oxidative damage, ensuring they can effectively perform their defensive roles. Prolonged oxidative stress has been shown to impair leukocyte activity, contributing to the development of inflammatory and autoimmune disorders [24]. Furthermore, biotin has been identified as a modulator of immune responses, with evidence suggesting it may influence leukocyte differentiation and function, potentially enhancing their ability to combat oxidative stress and maintain immune homeostasis [25]. Another study showed that ST in general reduced OS and anxiety levels while enhancing the antioxidant capacity in the testes of larger mice [26]. This study aimed to investigate the antioxidant capabilities of BS and the protective effects of ST against OS in male rats. We evaluated various biochemical parameters, oxidative/antioxidative markers, and testicular histology in male rats.

## 2 Materials and methods

### 2.1 Animals and experimental design

**Fig. 1:**
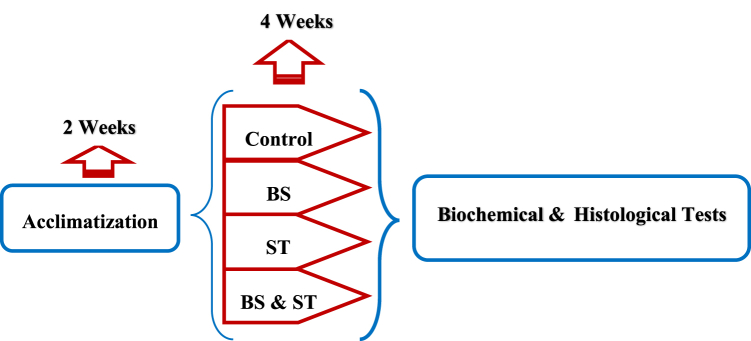
Experimental design of the study.

Forty adult male rats weighing 200–250 g were obtained from the animal house of the Hamadan University of Medical Sciences. They were housed in groups of five per cage and acclimatized to their new surroundings. The rats were then divided into the following experimental groups (n = 10 per group): 1) Control group (receiving a standard diet), 2) BS group (receiving BS (IRAN DARU Co., Iran) at 10 mg/kg/bw via oral gavage once a day for 28 days [27]), 3) ST group (performing ST for 28 days), and 4) BS + ST group (receiving BS (10 mg/kg/bw) via oral gavage once a day and perforing ST for 28 days. All animal studies were performed following the guidelines of Institutional Animal Care and Use approved by the Ethics Board of the Shiraz University of Agribusiness. Fig. 1 depicts the experiment design.

Before the experiments, the animals were separated for a week to get used to their environment. They were kept in a room with wire cages under a 12/12 light/dark cycle at an ambient temperature of 21 °C. All animals were fed with standard food and drank tap water throughout the experiment.

### 2.2 Swimming training

The animals underwent a 4-week ST regimen in a barrel-shaped tank measuring 148 cm × 50 cm, filled with temperature-controlled water (30–32 °C). The swimming program consisted of two phases: 1) an adaptation phase (water depth: 5 cm, 10 min) and 2) the ST phase (water depth: 5–15 cm, two or three sessions of 10 min each) [28].

### 2.3 Biochemical assays

Following the experiment, the rats were anesthetized using ketamine (100 mg/kg, Behbod Darou, Tehran, Iran) and xylazine (10 mg/kg, Alfasan, Woerden, Netherlands). Subsequently, their hearts were excised to obtain blood samples [29]. After coagulation of the blood, the test tubes were centrifuged for 15 min at 3000 rpm without any anticoagulant added. However, separating the blood serum proved to be challenging after removing the cylinder from the shaft. The samples were stored at −20 °C in the freezer until the measurement of follicle-stimulating hormone (FSH), Luteinizing Hormone (LH), and Testosterone (T) levels. Standard kits and the Enzyme-Linked Immunosorbent Assay (ELISA) method were employed in the laboratory to determine the concentrations of FSH, LH, and T in the blood serum of the animals [30]. The serum levels of triglycerides (TG), cholesterol (CHO), low-density lipoprotein (LDL), and high-density lipoprotein (HDL) were measured to evaluate the lipid profile, as dyslipidemia is often associated with oxidative stress and metabolic dysfunction, both of which can impact reproductive health [31]. Liver enzyme activities, including alanine transaminase (ALT), aspartate transaminase (AST), and alkaline phosphatase (ALP), were assessed to investigate potential effects of BS and ST on liver function, which plays a crucial role in lipid metabolism and overall homeostasis [32]. These markers were measured in blood serum samples following methods described in a previous study [33]. These assays were conducted to explore the combined effects of BS and ST on these markers, providing insights into their potential therapeutic benefits for metabolic regulation and reproductive health.

### 2.4 Measurement of the malondialdehyde (MDA) and glutathione (GSH) levels

On the day of analysis, testicular tissues were retrieved from the freezer and homogenized in ice-cold Phosphate-Buffered Saline (PBS) at 12,000 rpm for 1–2 min to create a 10 % homogenate (IKA, Germany). The supernatant was obtained by centrifuging the tissue homogenates at 5000 rpm for 30 min at +4 °C.

The analysis of MDA was conducted following the method outlined by Uchiyama and Mihara [34]. The evaluation of GSH levels was performed using Ellman's method [35].

### 2.5 Morphological and histopathological analysis

The weight of the right testis was documented after the removal of the rats’ testicles. Histological examinations were conducted on the left testis [29].

Following dehydration and embedding, the tissue (testis and epididymis) was sliced at a thickness of 5 μm and stained with hematoxylin and eosin (H&E) stain. The sections were examined, and images were captured using light microscopy at × 100 and × 400 magnifications. Twenty circular seminiferous tubules from each testis were chosen for histological assessment [36]. The mean of two diameters perpendicular to each section of the seminiferous tubules was computed.

Ten circular cross-sections of the seminiferous tubules were selected from each rat to measure the tubular diameter (TD). Then, two perpendicular diameters of each cross-section of seminiferous tubules were calculated at × 400 magnification. Additionally, within each cross-section of the seminiferous tubule, the height of the germinal epithelium was assessed in four equal segments, and the average was computed [37].

Sperm count was determined using a hemocytometer. To elaborate, the cauda epididymis of each rat was meticulously isolated, minced, and placed in 5 ml of physiological saline for 30 min at 37 °C to allow sperm to exit the tubules. Sperm motility was quantified as the percentage of motile sperm and assessed using a standard microscope at × 400 magnification. The total sperm count was calculated by enumerating the sperm count per milliliter [38,39].

### 2.6 Statistical analysis

Statistical analysis was conducted using SPSS 26. The Kolmogorov-Smirnov test was applied to indicate the normal distribution of data. A one-way ANOVA followed by Tukey's post hoc test was used for all statistical comparisons, with a significance level set at p < 0.05.

## 3 Results

### 3.1 Blood parameters

The results of this study showed that both the BS (P < 0.05) and BS + ST (P < 0.01) groups had significantly lower levels of CHO, TG, LDL, ALT, AST, and ALP compared to the control group (Table 1).

**Table 1:** Effect of different experimental treatments on biochemical parameters in male rats.

Biochemical Parameter	Control	BS	ST	BS & ST
Cholesterol (mg/dl)	94.30 ± 1.04	90.20 ± 0.84∗	91.10 ± 0.73	88.10 ± 1.01∗∗
Triglyceride (mg/dl)	82.60 ± 0.56	79.50 ± 0.61∗	79.70 ± 0.74∗	70.40 ± 0.88∗∗
LDL (mg/dl)	22.70 ± 0.55	20.90 ± 0.27∗	21.50 ± 0.34	19.20 ± 0.32∗∗
HDL (mg/dl)	35.90 ± 0.99	40.50 ± 34∗	39.40 ± 0.84	67.30 ± 1.33∗∗
ALT (U/L)	51.10 ± 1.60	46.10 ± 0.43∗	49.60 ± 0.99	44.70 ± 0.94∗∗
AST (U/L)	117.70 ± 0.59	115.20 ± 0.75	116.20 ± 0.64	114 ± 0.71∗∗
ALP (U/L)	123.70 ± 1.68	117.90 ± 0.60∗	120.50 ± 0.40	101.60 ± 1.35∗∗

Data are expressed as mean ± SEM (n = 10/group). Values having different superscripts are significantly different from the control group (∗p < 0.05) (∗∗p < 0.01).

Table 2 presents the mean increase in hormone levels. Hormonal analysis demonstrated a significant variance in the T levels of the BS + ST group compared to the other groups (P < 0.05). T levels in the BS + ST group (P < 0.05) were higher than in the control group.

**Table 2:** Effect of treatments on LH, FSH, and testosterone levels (ng/ml) of the experimental rats.

Hormonal Parameter	LH	FSH	Testosterone
Control	0.31 ± 0.02	0.18 ± 0.00	0.57 ± 0.42
Biotin	0.38 ± 0.02	0.18 ± 0.00	0.73 ± 0.03∗
Swimming	0.32 ± 0.02	0.18 ± 0.00	0.62 ± 0.04
Biotin & Swimming	0.42 ± 0.02∗	0.19 ± 0.00∗	0.76 ± 0.03∗∗

Data are expressed as mean ± SEM (n = 10/group). Values having different superscripts are significantly different from the control group (∗p < 0.05) (∗∗p < 0.01).

According to Table 3, GSH levels significantly increased in the BS, ST (p < 0.05), and BS + ST (p < 0.01) groups, whereas MDA levels significantly decreased in these experimental groups (p < 0.05 for all).

**Table 3:** Effect of experimental groups on antioxidants factors (n mol/g wet tissue) in serum of male rats.

antioxidants factors	MDA	GSH
Control	844.70 ± 1.13	649.20 ± 1.73
Biotin	840.30 ± 1.05∗	654.80 ± 0.97∗
Swimming	840.40 ± 0.70∗	654.50 ± 0.76∗
Biotin & Swimming	839.90 ± 1.03∗	656.80 ± 1.19∗∗

Data are expressed as mean ± SEM (n = 10/group). Values having different superscripts are significantly different from the control group (∗p < 0.05), (∗∗p < 0.01).

### 3.2 Morphological and histological findings

**Fig. 2:**
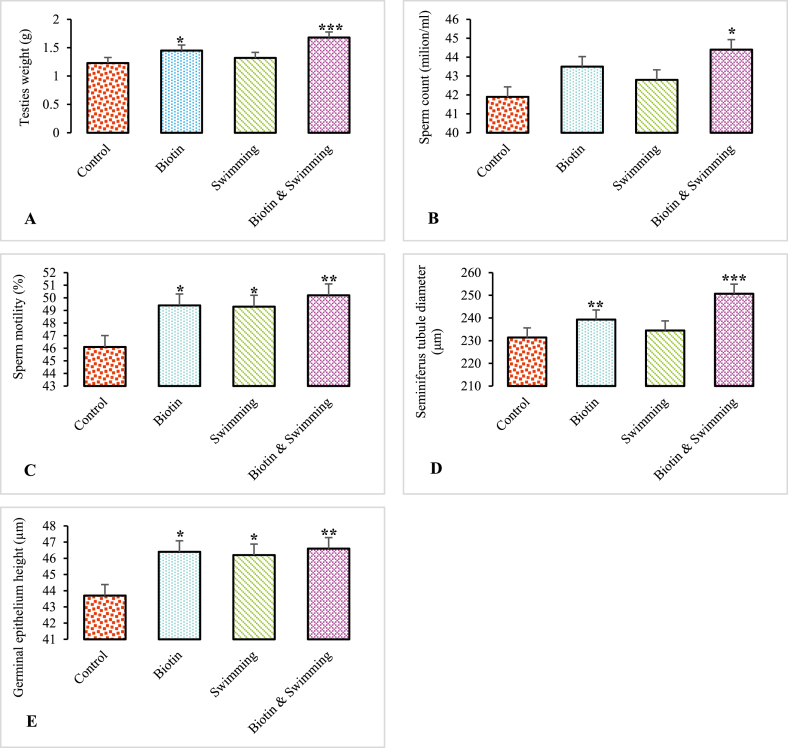
Effect of experimental treatment on testis weight (g) **(A)**, sperm count (million/ml) **(B)**, sperm motility (%) **(C)**, seminiferous tubule diameter (μm) **(D),** and germinal epithelium height (μm) **(E)**. The number of sperms increased significantly in the BS + ST group. ∗p < 0.05, ∗∗p < 0.01 and ∗∗∗p < 0.001, ∗ Denotes significant differences between control and experimental groups.

According to Fig. 2A and D), the testis weight (g) and TD (μm) significantly increased in the BS + ST group compared to the control group (p < 0.001). Germinal epithelium height (m) and sperm motility (%) were significantly higher in the BS + ST group than in the control group (p < 0.01) as shown in Fig. 2C and E. The BS group also displayed a significant difference (p < 0.01) from the control group concerning the TD (μm). Sperm motility (percent) and germinal epithelium height were significantly higher in the BS group (p < 0.05) than those in the control group. In addition, testis weight (g) in the BS group and sperm count in the BS + ST group were significantly different from those of the control group (p < 0.05; Fig. 2A and B).

**Fig. 3:**
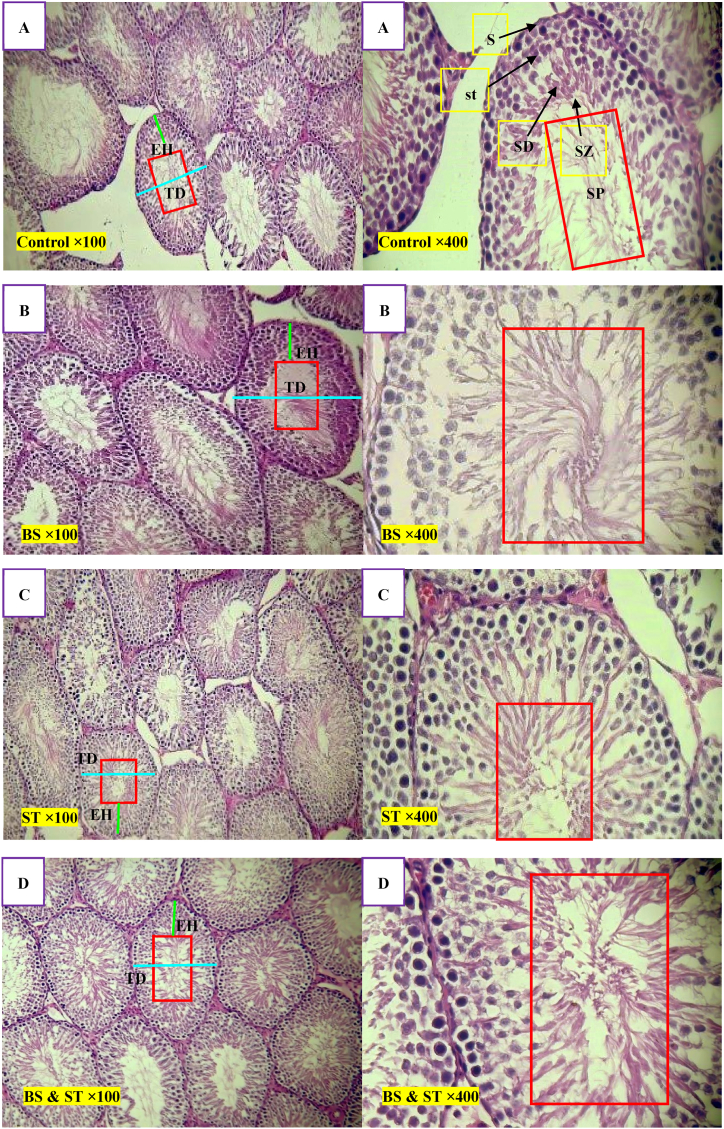
H&E (Hematoxylin and eosin) staining of rat testis (X = 100 (left), 400 (right) respectively); **(A)**, Control; **(B)**, BS: Biotin supplementation group; **(C)**, ST: Swimming Training group; **(D)**, BS & ST: Biotin supplementation with Swimming Training group. Blue lines indicate the diameter of the seminiferous tubule (TD), green lines indicate the epithelial height (EH) of the seminiferous tubule, and all red rectangles indicate the seminiferous lumen containing spermatozoa. The BS and BS & ST groups show a good appearance of testicular tubules containing cells of the spermatogenic series and lumen with spermatozoa (SZ). There is also the usual form of the germinal epithelium, spermatogonia (S), primary spermatocytes (st), and rounded spermatids (SD) in BS & ST rats. However, the ST group shows significantly reduced sperm density (SP) within the lumen, and photomicrographs of testis from this group show an increase in the interstitial space and thrombosis, degenerated spermatogenic epithelium series and interstitial cells, deteriorated spermatogonia and primary spermatocytes with karyorrhexis, complete absence of spermatids, destruction (depletion) in the germinal epithelium, an incomplete spermatogenic series, and fewer elongated spermatids, with interstitial edema. Scale bar = 100 μm.

Fig. 3 illustrates the relative variances in histological parameters due to experimental treatments. The results of the histological investigation revealed no significant alterations in any histological parameters within the control (Fig. 3A) and sham groups. On the other hand, the study found that biotin supplementation (Fig. 3B) alone or in combination (biotin supplementation + swimming training) (Fig. 3D) with swimming training (Fig. 3C) had positive effects on testicular health in rats. These findings suggest that biotin supplementation may be beneficial for maintaining testicular health, especially when combined with exercise.

## 4 Discussion

The correlation between male infertility and OS is extensively recognized and substantiated in current literature, with around 30 % of male infertility cases categorized as idiopathic [5]. Approximately 30 %–80 % of male subfertility cases are linked to the impacts of OS-induced damage, while 20 %–40 % of individuals facing infertility demonstrate significantly higher levels of ROS in their semen compared to fertile males [40]. Because of the abundant presence of ROS-inhibiting antioxidants in sperm and the elevated levels of unsaturated fatty acids within sperm structure, scientists posit that sperm is more vulnerable to OS compared to other cells [41]. Free radical-induced OS significantly impacts the alteration and fragmentation of sperm DNA, as well as the generation and proliferation of abnormal sperm, leading to a reduction in sperm count [42]. Meta-analytical results suggest that antioxidants offer a positive impact on individuals with varicocele. A meta-analysis of six studies encompassing 576 participants showcased a significant enhancement in sperm concentration, progressive and total motility, as well as normal morphology in those who underwent antioxidant therapy compared to the control group, three months after varicocelectomy [43]. In a recent meta-analysis, significant improvements were observed in sperm concentration, progressive and total motility, and seminal volume in varicocele patients who did not undergo surgical intervention, after receiving oral antioxidant supplements [44]. In a prospective study comprising 50 individuals with idiopathic infertility and 35 fertile controls, the influence of CoQ10 on conventional sperm parameters and antioxidant markers in seminal fluid was evaluated. The research demonstrated improvements in total and progressive motility, along with alterations in OS levels [45]. OS, if not countered by an antioxidant response, leads to oxidative damage, with MDA serving as a recognized biomarker of such damage [46]. As depicted in Table 3, the examination of MDA levels in the experimental groups unveiled a notable increase in the control group, indicating that the rodents were experiencing OS. Remarkably, LPO damage is presently considered the primary cause of testicular dysfunction [47]. Our experimental findings indicate that the combination of BS and ST mitigates OS, as evidenced by a reduction in MDA levels and an increase in GSH levels, highlighting its antioxidant potential. In this regard, the main justification for infertility lies in excessive generation of ROS or diminished cellular proliferation in semen, leading to OS and consequent reductions in sperm motility, increased sperm loss, and DNA fragmentation [48]. A recent study identified BS as an exogenous antioxidant due to its potent antioxidant properties [49]. Vitamins are essential for the maturation of SZ. For example, a deficiency in vitamin B12, a water-soluble nutrient, was observed to induce histological alterations in rodent testicles, resulting in sperm aplasia [50]. A deficiency in L-ascorbic acid can lead to disruptions in sperm production [51]. Studies have demonstrated that Vitamin C supplementation can safeguard human spermatogenesis germ cells from OS [52]. In addition, a significantly reduced level of GSH was observed in the testes of animals in the control group, suggesting that ROS-mediated testicular damage disrupts cellular reinforcement mechanisms. The dysfunction in T biosynthesis can also be attributed to decreased levels of GSH in the testes [53]. GSH, a tripeptide containing thiols, is present in almost all cells and plays a vital role in the cellular antioxidant defense system [53]. It serves as a significant endogenous antioxidant generated by cells, playing an active role in counteracting ROS and aiding in the conservation of exogenous antioxidants such as vitamins C and E in their unoxidized forms [54]. As a result, ROS production rises as GSH levels decline [53]. However, swimming for two weeks significantly reduced testicular size [55]. Moderate-intensity swimming enhanced testicular cancer-preventive status by up-regulating levels of testicular GPX, GSH, Turf, and Feline [26,56]. Also, the increase in OS caused by swimming could disrupt the male reproductive system [19].

Biotin also acts as an antioxidant, safeguarding germ cells involved in spermatogenesis from OS. This vitamin enhances reproductive success in animals by supporting ovulation and improving pregnancy and fertilization rates [57]. Biotin is hypothesized to influence the hypothalamic-pituitary-gonadal (HPG) axis, potentially regulating gonadotropins such as LH and FSH. A deficiency in biotin could disrupt testicular function, altering LH and FSH secretion patterns. Within the testicles, Leydig cells, which synthesize steroid hormones, are regulated by LH from the anterior pituitary gland via the hypothalamic-pituitary-testicular (HPT) axis [58]. Thus, biotin may boost T production by acting directly on testicular steroidogenic cells.

Spermatogenesis is highly dependent on T, which drives cell division and growth. Research by Paulose et al. (1989) [59] demonstrated that biotin-deficient rats showed decreased T levels in both their testicles and bloodstream. This highlights the potential role of biotin, alongside T and FSH, in supporting the function of Leydig cells, Sertoli cells, and peritubular cells. Biotin may also be crucial for testicular development, including meiosis and sperm maturation [17]. The observed increase in FSH and LH levels can be attributed to the synergistic effects of BS and ST in reducing oxidative stress, as indicated by increased GSH and decreased MDA levels. Oxidative stress has been shown to impair the HPG axis, leading to hormonal imbalances. By alleviating oxidative stress, BS and ST likely restored the normal functioning of the HPG axis, thereby contributing to the improved hormonal profile observed in our study.

Biotin, a nutrient whose impact on testicular function has been explored in rodents [60], is crucial for testicular development, including processes like meiosis and sperm maturation, as well as for facilitating normal interactions among Leydig, Sertoli, and peritubular cells, alongside T and FSH [17]. Another study found that extremely high doses of biotin can affect sperm quality, spermatogenesis, and hepatic and testis morphology [14]. Conversely, biotin was found to enhance T production in testis-derived cells in mice [14]. In males, supplementing the diet with micronutrients and cell enhancers may enhance sperm viability, motility, and morphology by regulating the system that governs sperm impedance and improving hormonal balance [61]. A healthy lifestyle emphasizes consuming a balanced, nutritious diet and engaging in physical activity [62].

Given that swimming is a prevalent ET model and aligns with the natural behavior of rodents, it was selected as a suitable endurance ET model in this research [63]. Swimming reduces OS and inflammation in people with chronic colitis [64], and decreases OS in the liver [65]. Two weeks of swimming notably decreased testicular size [55]. The mitochondrial layer of the testis is rich in polyunsaturated lipids [66]. Furthermore, the testicles are highly vulnerable to peroxidative injury. Studies have demonstrated that ET can decrease LPO and boost antioxidant enzyme activity in crucial animal tissues [66]. Certain researchers posit that ET elevates oxygen consumption by up to 10–20 times, leading to increased ROS production in cells and tissues, thereby inducing OS that hastens damage [67]. Regardless of the cause, OS affects testicular blood flow and vascular tone as well as the oxidant/antioxidant balance. A significant decrease in blood flow, as well as direct oxidative harm to the testicular tissue, affects T levels. Decreased blood supply to Leydig cells or the direct negative effect of ROS on Leydig cells could be responsible for the decline in T production. Swimming at a moderate intensity for eight weeks resulted in a significant increase in T synthesis, serum Luteinizing Hormone (LH) levels, and sperm quality in mice fed a high-fat diet [26]. Moderate swimming ET can significantly protect sperm vitality and T production from testicular OS and inflammation [68].

This study explores the role of BS and ST in improving lipid profiles and lowering liver enzyme activities in blood serum samples (Table 1). The results suggest that BS and ST offer therapeutic benefits for lipid metabolism and liver function by reducing ALT, AST, and ALP levels. The mechanism through which BS regulates lipid homeostasis is primarily attributed to its involvement in biotin-dependent carboxylases [69]. These include key enzymes such as acetyl-CoA carboxylase (ACC), propionyl-CoA carboxylase (PCC), 3-methylcrotonyl-CoA carboxylase (MCC), geranyl-CoA carboxylase (GCC), and long-chain acyl-CoA carboxylase (LCC), along with smaller organic compounds such as pyruvate carboxylase (PC) and urea carboxylase [70]. Among these, ACC plays a pivotal role in fatty acid biosynthesis, with two isoforms: ACC-1 and ACC-2. ACC-1 is crucial for the synthesis of long-chain fatty acids in the liver and adipose tissues, while ACC-2 is predominantly active in the heart, skeletal muscles, and liver [71]. PCC, on the other hand, facilitates the catabolism of cholesterol, odd-chain fatty acids, and specific amino acids [70]. Prior research has shown that BS supplementation reduces serum total cholesterol and triglyceride concentrations in rats, while liver enzyme activities (ALT and AST) remain unchanged across treatments [69]. Similarly, a study demonstrated that dietary BS supplementation in mice significantly decreased blood triglyceride levels [72].

Our findings indicate that adhering to a suitable moderate exercise regimen can be beneficial for preserving fertility. It may be advantageous to incorporate vitamins as antioxidants alongside diverse exercises in your routine to enhance antioxidant levels. To gain a deeper insight into how BS and ST treatments influence testicular function, further experiments are necessary.

## 5 Conclusion

This is the first study to demonstrate that BS along with ST plays a role in male reproductive processes. The selection of these specific experimental treatments was driven by the study's objective to unveil the novel roles of BS and ST in male reproduction. While our research has demonstrated the critical role of biotin in spermatogenesis, additional investigations are warranted to assess its potential and effectiveness in clinical trials. It is crucial to conduct further studies involving a more extensive sample size of animals encompassing diverse species. Healthcare practitioners are recommended to stay informed about the latest advancements in biotin research due to its importance in human health. Similarly, researchers are urged to explore biotin studies to leverage the promising opportunities stemming from recent progress in this field.

## CRediT authorship contribution statement

**Shadi Almasi:** Methodology, Formal analysis, Data curation, Conceptualization. **Mohammad Reza Jafarzadeh Shirazi:** Visualization, Validation, Supervision, Methodology, Funding acquisition. **Mohammad Reza Rezvani:** Software, Methodology, Investigation, Funding acquisition, Formal analysis. **Mahdi Ramezani:** Validation, Software, Project administration, Formal analysis, Data curation. **Iraj Salehi:** Validation, Resources, Project administration, Data curation, Conceptualization. **Sasan Javid-Moghadam:** Software, Methodology, Formal analysis. **Alireza Komaki:** Writing – review & editing, Writing – original draft, Supervision, Methodology, Formal analysis, Conceptualization.

## Data availability statement

Data will be made available on request.

## Funding statement

This research was supported by 10.13039/501100005071Shiraz University (Grant No.: 0gcb3m148075).

### Ethical Approval

All animal procedures were performed in accordance with the institutional and national guidelines (ARRIVE guidelines) for animal care and approved by the Animal Research Ethical Committee of the Shiraz University of Agriculture (Ethics Code: IR.SHZU.REC. 0gcb3m148075).

## Declaration of competing interest

The authors declare that they have no known competing financial interests or personal relationships that could have appeared to influence the work reported in this paper.
